# Cnidarian phylogenetic relationships as revealed by mitogenomics

**DOI:** 10.1186/1471-2148-13-5

**Published:** 2013-01-09

**Authors:** Ehsan Kayal, Béatrice Roure, Hervé Philippe, Allen G Collins, Dennis V Lavrov

**Affiliations:** 1 Dept. Ecology, Evolution, and Organismal Biology, Iowa State University, 50011, Ames, Iowa, USA; 2 Department of Invertebrate Zoology, National Museum of Natural History, Smithsonian Institution, 20013-7012, Washington, DC, USA; 3 Dept. Biochimie, Fac. Médecine, Université de Montral, Pavillon Roger-Gaudry, C.P. 6128, Succ. Centre-Ville, H3C 3J7, Montral, QC, Canada; 4 National Systematics Laboratory of NOAA’s Fisheries Service, National Museum of Natural History, MRC-153, Smithsonian Institution, PO Box 37012, 20013-7012, Washington, DC, USA

**Keywords:** Cnidaria, Medusozoa, Acraspeda, Anthozoa, mito-phylogenomics

## Abstract

**Background:**

Cnidaria (corals, sea anemones, hydroids, jellyfish) is a phylum of relatively simple aquatic animals characterized by the presence of the cnidocyst: a cell containing a giant capsular organelle with an eversible tubule (cnida). Species within Cnidaria have life cycles that involve one or both of the two distinct body forms, a typically benthic polyp, which may or may not be colonial, and a typically pelagic mostly solitary medusa. The currently accepted taxonomic scheme subdivides Cnidaria into two main assemblages: Anthozoa (Hexacorallia + Octocorallia) – cnidarians with a reproductive polyp and the absence of a medusa stage – and Medusozoa (Cubozoa, Hydrozoa, Scyphozoa, Staurozoa) – cnidarians that usually possess a reproductive medusa stage. Hypothesized relationships among these taxa greatly impact interpretations of cnidarian character evolution.

**Results:**

We expanded the sampling of cnidarian mitochondrial genomes, particularly from Medusozoa, to reevaluate phylogenetic relationships within Cnidaria. Our phylogenetic analyses based on a mitochogenomic dataset support many prior hypotheses, including monophyly of Hexacorallia, Octocorallia, Medusozoa, Cubozoa, Staurozoa, Hydrozoa, Carybdeida, Chirodropida, and Hydroidolina, but reject the monophyly of Anthozoa, indicating that the Octocorallia + Medusozoa relationship is not the result of sampling bias, as proposed earlier. Further, our analyses contradict Scyphozoa [Discomedusae + Coronatae], Acraspeda [Cubozoa + Scyphozoa], as well as the hypothesis that Staurozoa is the sister group to all the other medusozoans.

**Conclusions:**

Cnidarian mitochondrial genomic data contain phylogenetic signal informative for understanding the evolutionary history of this phylum. Mitogenome-based phylogenies, which reject the monophyly of Anthozoa, provide further evidence for the polyp-first hypothesis. By rejecting the traditional Acraspeda and Scyphozoa hypotheses, these analyses suggest that the shared morphological characters in these groups are plesiomorphies, originated in the branch leading to Medusozoa. The expansion of mitogenomic data along with improvements in phylogenetic inference methods and use of additional nuclear markers will further enhance our understanding of the phylogenetic relationships and character evolution within Cnidaria.

## Background

**Figure 1:**
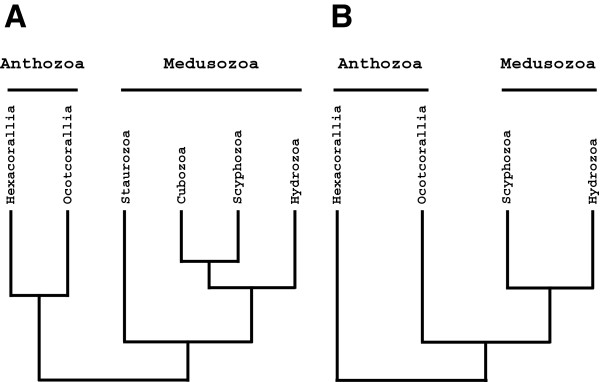
**Alternative hypotheses of the cnidarian tree of life. A**. Current view of cnidarian evolutionary history based on rRNA phylogenies. **B**. Hypothesis of cnidarian relationships obtained using mitochondrial protein coding genes.

The phylum Cnidaria encompasses five classes: Anthozoa, Cubozoa, Hydrozoa, Scyphozoa, and Staurozoa. Anthozoa is the most speciose of these classes and is further subdivided into two diverse subclasses Hexacorallia (hard corals and sea anemones) and Octocorallia (soft corals, sea pens, and gorgonians) [1,2]. The remaining four classes – Cubozoa (box jellies or sea wasps), Hydrozoa (hydras, hydroids, hydromedusae, and siphonophores), Scyphozoa (true jellyfish), and Staurozoa (stalked jellyfish) – are united in the subphylum Medusozoa. Cubozoa (Werner 1975) and Staurozoa (Marques and Collins 2004) were originally included in the class Scyphozoa (Götte 1887), but each has been promoted to the status of class, leaving three orders Coronatae, Rhizostomeae and Semaeostomea in Scyphozoa [1]. Recent progress in understanding cnidarian phylogeny, particularly efforts from the Cnidarian Tree of Life (CnidToL) project (cnidtol.com), based on analyses of rRNA data, have yielded a relatively widely accepted view of cnidarian relationships (Figure 1A). Nevertheless, these relationships are hypothetical and subject to tests with alternative datasets, and questions about the relationships among and within the major cnidarian taxa remain.

For instance, monophyly of Anthozoa is supported by numerous analyses of rRNA data [3-6], although only one of them [7] included a dense sampling of both anthozoan and medusozoan taxa. However, studies based on mitochondrial DNA data suggest that Anthozoa is paraphyletic, with octocorals forming a sister group relationship with medusozoans [8-11] (Figure 1B). The monophyly of Medusozoa has not been challenged, but relationships within it remain somewhat contentious. As an example, although traditionally box jellies (Cubozoa) were considered to be closely related to true jellyfish (Acraspeda; Gegenbaur 1856; in accordance with rRNA-based analyses), some studies have suggested the groupings of [Cubozoa + Staurozoa] and [Hydrozoa + Scyphozoa] [12,13]. Furthermore, the early branching position of Staurozoa, as sister group to the remaining medusozoans only received moderate support in rRNA studies [14].

Even within the major cnidarian taxa, phylogenetic hypotheses remain to be assessed with independent datasets. Some studies have nested the monophyletic rhizostome jellyfish within a paraphyletic Semaeostomeae [7,14-16], a view supported by the most recent phylogenetic study using rRNA sequences [17]. Other relevant findings include the sister group relationships between Trachylina and Hydroidolina within Hydrozoa [14], paraphyletic “Filifera” (Kühn 1913) within Hydroidolina [18,19]; monophyletic stony corals (Scleractinia) within Hexacorallia [20,21] in opposition to earlier studies [22]; and, two robust clades Carybdeida and Chirodropida composing box jellyfish (Cubozoa) [23]. In addition, rDNA-based studies have also exposed some disparities between classical taxonomy and molecular phylogenies for some groups [24] and were unable to resolve phylogenetic relationships within others, e.g. Hydroidolina [14,19] and alcyonacean octocorals [24]. Thus, additional markers are necessary to achieve a better understanding of cnidarian relationships.

Resolving phylogenetic relationships for the phylum Cnidaria is a prerequisite for the reconstruction of the evolutionary history of key morphological novelties in this group, e.g. life history and morphological characters [14,18,25], medusan morphospace and swimming ability [26], the evolution of toxicity in cubozoans [23], the origin and evolution of reef-building corals [20,21], and mitochondrial genome structures [27]. One critical character in cnidarian evolution is the ancestral state of the adult life stage, polyp or medusa, which has been a matter of controversy for over a century [14,28]. All anthozoans (hexacorals and octocorals) lack a free-living medusa stage, while cubozoans and most scyphozoans contain both polyp and medusa stages. Hydrozoans display the widest range of diversity in their life cycle, with the absence of a sessile polyp in Trachymedusae (which may be diphyletic [29]) and many species of Narcomedusae, and a highly reduced or absent medusa in other lineages (e.g. some clades within Leptothecata and Aplanulata, including the freshwater hydras (Hydridae)) [1,7,18,30,31]. While some earlier studies have proposed the medusa form has been lost in Anthozoa, the current view holds the medusa is an apomorphy (derived character) for Medusozoa [1,14]). Consequently, it is generally considered that (1) a sessile polyp-like form was the ancestral adult form in the phylum Cnidaria and, (2) an adult pelagic medusa phase evolved (one or several times) in Medusozoa [1,14]. However, the latter view has only slight phylogenetic support in the currently accepted cnidarian phylogeny, where the most parsimonious scenario involves the gain of the polyp form in the ancestral Cnidaria and of the medusa form in Medusozoa. Alternatively, both the polyp and the medusa forms could have been acquired in the ancestral cnidarian, and the medusa form subsequently lost in the branch leading to Anthozoa. The multiple losses of the medusa stage in different medusozoan lineages suggest that this character is indeed evolutionary labile.

Medusozoa (sometimes referred to as Tesserazoa) is supported by a combination of characters both morphological (e.g. presence of an adult pelagic stage, cnidocils (cilia of cnidocytes lacking basal rootlets), and microbasic eurytele nematocysts) and molecular (e.g. rDNA, mitochondrial protein genes, linear mitochondrial DNA) [1,14,28]. Similarly, several morphological characters have been suggested as synapomorphies for Anthozoa, including the presence of an actinopharynx (a tube leading from the mouth into the coelenteron), mesenteries and possibly siphonoglyphs (ciliated grooves longitudinally extending along the pharynx), although the latter are absent in some anthozoan lineages [1]. As noted above, conflicting evidence exists about the monophyly of Anthozoa, being supported by rRNA data [3-6] (Figure 1A) and contradicted by mitochondrial genome DNA data [8-11] (Figure 1B). This alternative phylogenetic hypothesis, if true, would necessitate reinterpretation of morphological characters shared by anthozoans as symplesiomorphies (retained from the common ancestor) rather than synapomorphies.

Mitochondrial DNA (mtDNA) is a popular molecular marker for understanding the phylogenetic relationships in animals. Recent technological advances in sequencing complete mtDNA sequences have provided easier access to mitogenomic data for phylogenomic studies [32-34]. Some suggested advantages of mtDNA over nuclear DNA (nDNA) in phylogenetics are the asserted orthology of all genes [35] and the small genome structure being relatively conserved, which provides additional characters such as gene order (considered as Rare Genetic Changes or RGC) [36,37]. Despite some limitations, mtDNA-based phylogenetic trees are considered valid proxies of the evolutionary history within and between most metazoan groups. In non-bilaterian animals, recent increase in the number of completely sequenced mtDNAs has helped to resolve deep phylogenetic nodes within sponges [9,38] and Hexacorallia within Cnidaria [22,39]. Yet, the very poor sample size of medusozoan taxa in previous studies raises some question about the validity of phylogenetic results based on them. Indeed, it is known that inadequate taxon sampling and systematic errors can override genuine phylogenetic signal, resulting in flawed phylogenetic reconstructions [40,41].

We assembled a more taxonomically balanced mitogenomic dataset to investigate the evolutionary history of cnidarians. Our dataset contains newly published mitochondrial sequences from 24 representative species of all medusozoan classes, including three orders of Scyphozoa, both orders of Cubozoa, and six out of the nine orders of Hydrozoa [27]. We also included sequences from two octocoral orders Penatulacea and Helioporacea, and two hexacoral orders Antipatharia and Ceriantharia. Our analyses suggest that the paraphyly of Anthozoa does not result from poor taxon sampling. We also found the groupings [Discomedusae + Hydrozoa] and [Coronatae + [Cubozoa + Staurozoa]], contradicting the current rDNA-based phylogenetic hypothesis within Medusozoa.

## Results

### Additional mitogenomes for Hexacorallia and Octocorallia

For this study, we amplified and sequenced the complete mtDNA of the black coral *Cirripathes lutkeni* (20,448 bp), the sea pens *Renilla muelleri* (18,643 bp) and *Stylatula elongata* (18,733 bp), and the alcyonarian *Sinularia peculiaris* (18,742 bp) as described earlier [27] and partial mt sequences for the cerianthid *Ceriantheopsis americanus* and the octocoral *Heliopora coerulea*. All three complete octocoral mt-genomes have the same genome organization as that of *Sarcophyton glaucum*. The mtDNA of *C. lutkeni* is similar to that of *Chrysopathes formosa*, but possesses an intron within *cox1* that harbors a HEG-like ORF, responsible for the larger genome size. Partial data from the mtDNA of *C. americanus* does not allow us to discuss the mitochondrial genome organization in Ceriantharia.

### Models of sequence evolution in our phylogenetic analyses

We evaluated how the site-heterogeneous CAT and CATGTR models perform on mitochondrial protein amino acid dataset (AliMG) compared to the reference site-homogeneous model GTR under BI by using cross-validation. According to pair-wise difference of log-likelihood scores, we found that the CATGTR model more accurately explained our data (330.78 +/− 40.071); the CAT model was the worst of the three models for our alignment (−111.61 +/− 63.337). For all the codon alignments, we found GTR to be the best-fit model according to the Bayesian Information Criterion (BIC), the corrected Akaike Information Criterion (AICc), and the Decision Theory Performance-Based selection (DT). We therefore used the GTR models as well as the Q-Matrix Mixture model (QMM) implemented in PB for all codon analyses.

### Phylogenetic relationships among cnidarian classes

**Figure 2:**
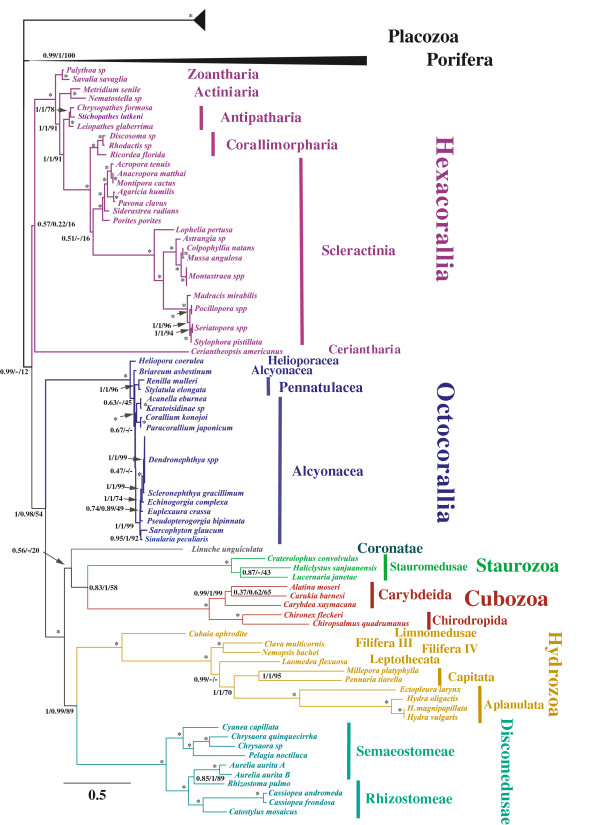
**Cnidarian phylogenetic hypothesis based on mitochondrial protein genes.** Phylogenetic analyses of cnidarian protein coding genes under the CATGTR model with PhyloBayes for the AliMG alignment (3111 positions, 106 taxa) created using the MUSCLE plug-in in Geneious and filtered using Gblock. Support values correspond to the posterior probabilities for the CATGTR(BI) and GTR(BI), and bootstraps for GTR(ML) analyses. Stars denote maximum support values. A dash denotes discrepancy between the results obtained when assuming different models.

We analyzed the phylogenetic relationships among cnidarian classes for both the amino acids and codon data under the Bayesian (BI) and the Maximum Likelihood (ML) frameworks. We also used two different models of sequence evolution (GTR and CATGTR) for Bayesian inferences on the amino acids alignment. For all our analyses, we decided to exclude sequences from bilaterian animals because they form long branches in mitochondrial phylogenomic trees that attract other long branches, resulting in Long Branch Attraction artifacts [42,43]. In the future, the inclusion of bilaterians in mtDNA-based phylogenies can be tested given better models of sequence evolution are available. We found maximum support for the monophyly of Medusozoa, Cubozoa, Staurozoa, Hydrozoa, and Discomedusae in all amino acid analyses (Table 1). In the analyses based on amino acid sequences, a few differences emerged when using two different models of sequence evolution, most of them limited to poorly supported branches. For instance, we found monophyletic Cnidaria (posterior probability PP = 1) and Hexacorallia (but with no support PP = 0.57), and the placement of the coronate *Linuche unguiculata* as the sister taxa to the clade [Cubozoa + Staurozoa] (PP = 0.56) under the CATGTR model (Figure 2). On the other hand, we found paraphyletic Cnidaria and Hexacorallia in all GTR trees, where the position of *Ceriantheoptsis americanus* was unstable (data not shown). We also observed that *L. unguiculata* was the first diverging medusozoan clade in GTR (BI) analyses but the sister taxa to [Cubozoa + Staurozoa] in GTR (ML) without support (bootstraps BV = 20). Removing the coronate *L. unguiculata* and those species with missing data (the blue coral *Heliopora coerulea* and the tube anemone *C. americanus*) did not impact cnidarian paraphyly under GTR (ML) model (Additional file 1: Figure S1).

**Table 1:** Support values for topologies in the mtDNA-based phylogeny of cnidarians

		**AliMG**	
Taxon	GTR-ML	GTR-BI	CATGTR
Cnidaria	12	0	1
Anthozoa	0	0	0
Hexacorallia	12	0.22	0.57
Antipatharia	100	1	1
Actiniaria	100	1	1
Corallimorpharia	100	1	1
Scleractinia	12	0	0.51
Zoantharia	100	1	1
Octocorallia	100	1	1
Alcyonacea	7	0	0.01
Pennatulacea	96	1	1
Medusozoa	100	1	1
Acraspeda	0	0	0
Cubozoa	100	1	1
Carybdeida	99	1	1
Chirodropida	100	1	1
Hydrozoa	100	1	1
Aplanulata	100	1	1
Capitata	95	1	1
Scyphozoa	0	0	0
Discomedusae	100	1	1
Rhizostomeae	11	0	0.14
Semaeostomeae	0	0	0
Staurozoa	100	1	1
Placozoa	100	1	1
Porifera	100	1	0.88
Homoscleromorpha	100	1	1
Demospongiae	100	1	1
Keratosa	100	1	1
Myxospongiae	100	1	1
marine Haplosclerida	100	1	1
Democlavia	100	1	1

Bootstraps values (for GTR-ML) and posterior probabilities (for GTR and CATGTR-BI) obtained for the AliMG alignment for several clades traditionally recognized in Cnidaria.

All analyses supported the clade [Octocorallia + Medusozoa] (CATGTR: PP = 1; GTR: PP = 0.98; BV = 54), although posterior probabilities (PP) were lower when GTR model was used (Figure 2). Within Medusozoa, we recovered the monophyly of Cubozoa, Staurozoa, and Hydrozoa with maximum support, but Scyphozoa [Coronatae + Discomedusae] was not recovered as a monophyletic group in any analyses (Table 1). The class Hydrozoa always grouped with Discomedusae (CATGTR: PP = 1; GTR: PP = 1 BV = 89), and Cubozoa was the sister taxon to Staurozoa (CATGTR: PP = 0.83; GTR: PP = 1 BV = 58). When we removed the single species of coronate, *L. unguiculata*, the support values for the clade [Cubozoa + Staurozoa] slightly increased (BV = 85), while support values for the clade [Hydrozoa + Discomedusae] decreased (BV = 66).

We also analyzed codon alignments from a subset of 75 species using the GTR and the QMM models under BI and the GTR model under the ML frameworks. It has been shown that codon usage bias can result in phylogenetic artifact [44]. In order to minimize the impact of codon usage bias on phylogenetic trees, we reanalyzed the codon dataset after removing the third codon position, as well as codons for arginine (AGR and CGN), leucine (CTN and ATH), and serine (TCN and AGY). Only the CodAliM75tx-argleuser3 dataset under GTR and the CodAliM75tx-argleuser3 and the CodAliM75tx-ser3 datasets under QMM model produced informative trees (Additional file 2: Figures S2 and Additional file 3: Figure S3), the other analyses resulting in the near absence of phylogenetic resolution. When some resolution was achieved, the codon analyses yielded similar results to those from the amino acid dataset. We found paraphyletic Anthozoa, with Medusozoa the sister taxon to Octocorallia (PP > 0.95) in all codon analyses, but monophyletic Cnidaria only in the QMM analysis of the CodAliM75tx-ser3 dataset (PP = 0.85, Additional file 3: Figure S3).

### Intra-class relationships

Within Discomedusae, our data reject the monophyly of Semaeostomeae and Rhizostomeae (Table 1) with the rhizostome *Rhizostoma pulmo* as the sister taxon to the ulmariid semaeostome *Aurelia aurita*, while uniting the semaeostome families Cyaneidae and Pelagiidae, as well as all the remaining rhizostomes. Cubozoa is divided into the two monophyletic clades Carybdeida and Chirodropida as found earlier [23], but our data do not resolve the relationships between the three carybdeids. In Hydrozoa, our analyses supported the dichotomy Trachylina (*Cubaia aphrodite*) – Hydroidolina (the rest of the hydrozoans) with high support values (PPs = 1; BVs = 100; Figure 2). The aplanulatan *Hydra* spp and *Ectopleura larynx* and the capitates *Millepora platyphylla* and *Pennaria disticha* formed a monophyletic clade (PPs = 1; BV = 70). The species *Clava multicornis* (Filifera III) and *Nemopsis bachei* (Filifera IV) also formed a monophyletic clade as the sister group to the rest of Hydroidolina with maximum support values. The position of the leptothecate *Laomedea flexuosa* was different between GTR and CATGTR analyses. The GTR trees placed Leptothecata as the sister taxon to the clade [Filifera III + Filifera IV] (PP = 1; BV = 86) similar to ML analyses using nuclear and mitochondrial rRNA genes with the GTRMIX model of sequence evolution [18]. By contrast, Bayesian analysis using the CATGTR model strongly supported the leptothecate hydrozoan as the sister taxon to the clade [Aplanulata + Capitata] (PP = 0.99).

Our data do not resolve the position of Ceriantharia (tube anemones). Given that our sequence for the tube anemone is incomplete and our sampling only includes one species from Ceriantharia, additional data from the group might provide some clues on the position of cerianthids. We recovered the currently accepted order-level relationships [20] for the remaining hexacorals in all our analyses (Figure 2). Zoanthidea (zoanthids) was the earliest diverging lineage, followed by Actiniaria (sea anemones), Antipatharia (black corals), and the clade [Corallimorpharia + Scleractinia]. The order Scleractinia (stony corals) was monophyletic in CATGTR analyses with low support values (PP = 0.51), but paraphyletic under GTR framework, where one clade of scleractinians was the sister taxa to corallimorphs (PP = 1;BV = 92). Finally, despite the addition of two new sequences from Pennatulacea (*Renilla muelleri* and *Stylatula elongata*) and partial data from *Heliopora coerulea*, our analyses did not resolve the phylogenetic relationships within Octocorallia.

### Testing evolutionary hypotheses

We used several phylogenetic tests to evaluate the support for traditional taxonomic hypotheses of our datasets under the ML framework. We used the Approximately Unbiased (AU) test, the Kishino-Hasegawa (KH) test, and the Shimodaira-Hasegawa (SH) test with the GTR + Γ model (Table 2). We found that the order Semaeostomeae is significantly rejected (AU = 0.01; KH = 0.02; SH = 0.18), but not the inclusion of staurozoan species as part of an extensive “Scyphozoa” clade (AU = 0.01; KH = 0.04; SH = 0.25). We also found that despite the absence of support for the monophyly of both Hexacorallia and Scleractinia in GTR trees, hypothesis tests do not reject these clades (Table 2). In addition, GTR-based analyses do not significantly reject the validity of Anthozoa (AU = 0.63; KH = 0.41; SH = 0.93), Scyphozoa (AU = 0.10; KH = 0.08; SH = 0.42), or the clade Acraspeda [Cubozoa + Scyphozoa] (AU = 0.80; KH = 0.59; SH = 0.99), even after removal of the long-branch coronate *L. unguiculata* (AU = 0.75; KH = 0.66; SH = 0.90; Additional file 4:Figure S4). By comparison, under BI we found no support for the validity of Anthozoa (PPs = 0; BV = 0), Acraspeda (PPs = 0; BV = 0), Scyphozoa (PPs = 0; BV = 0), and Semaeostomeae (PPs = 0; BV = 0) in any of our trees under both GTR and CATGTR models.

**Table 2:** The use of several statistical tests verifying the validity of some groups in cnidarians

		**AliMG**	
	**AU**	**KH**	**SH**
Cnidaria	0.27	0.15	0.86
Anthozoa	0.63	0.41	0.93
Hexacorallia	0.48	0.32	0.86
Scleractinia	0.25	0.17	0.53
Acraspeda*	0.80	0.59	0.99
Rhizostomeae	0.24	0.17	0.64
Scyphozoa	0.10	0.08	0.42
Scyphozoa + Cubozoa + Hydrozoa*	0.12	0.14	0.41
Scyphozoa + Cubozoa + Staurozoa*	0.04	0.01	0.27
Scyphozoa + Staurozoa*	0.01	0.04	0.25
Semaeostomeae	0.01	0.02	0.18

Probability values for the AU, KH and SH tests and BI values for several clades traditionally recognized in Cnidaria.

* similar results have been obtained for a smaller dataset that does not contain the sequences from the coronate *Linuche unguiculata*, the tube anemone *Ceriantheopsis americanus* and the blue octocoral *Heliopora coerulea*.

## Discussion

We reevaluated cnidarian phylogenetic relationships using a dataset of mitochondrial protein genes from a systematically more balanced sample of species, including cubozoans and staurozoans [27], which were absent in previous works [10,12]. Our cnidarian-rich dataset reduced one major source of potential error that could derive from biased and insufficient taxon sampling for the groups of interest. In our analyses, the site-heterogeneous model CATGTR recovered the monophyly of Cnidaria and Hexacorallia, both supported by molecular and morphological characters [12,45-49]. By contrast, these clades were paraphyletic in both Maximum Likelihood and Bayesian analyses based on the GTR model of sequence evolution. This is not surprising given that a main assumption underlying the GTR model, i.e. homogeneity of the substitution pattern across sites, is violated by most molecular data, rendering the correct capture of the phylogenetic signal present in our alignments more difficult [50]. The resulting topologies from CATGTR analyses differed significantly from the current consensus view of cnidarian phylogeny [1,2]. Using the best estimate as a working hypothesis for cnidarian relationships, we can propose a putative reconstruction of character evolution for the group.

### The mitogenomic point of view of cnidarian systematics

The current view on cnidarian phylogeny based on nuclear rRNA genes subdivides the phylum Cnidaria into Anthozoa and Medusozoa [1,2], but mitochondrial protein genes have consistently supported anthozoan paraphyly with Octocorallia being the sister taxon to Medusozoa [8-11]. It has been argued that the paraphyly of Anthozoa reported in earlier mitogenomic studies resulted from poor taxon sampling [2,10]. Here we show that paraphyletic Anthozoa does not result from unbalanced taxon sampling. In addition, a principal component analysis of the amino acid composition (Additional file 5: Figure S5) suggests that compositional bias is also an unlikely explanation. The amino acid composition of Hexacorallia is rather divergent, but not similar to those of the two outgroups (Porifera and Placozoa); in fact, the composition similarity between Octocorallia and the outgroups would favor the alternative possibility of Anthozoa paraphyly [Medusozoa + Hexacorallia], which is not observed in our analyses. To further test the possible impact of compositional bias, we analyzed our alignments using the CATGTR model with a Dayhoff recoding strategy, despite the fact that it implies a loss of signal resulting in increasing the stochastic error. Interestingly, Octocorallia remained sister-group of Medusozoa, even if the statistical support was reduced (data not shown).

We also analyzed codon alignments using the GTR and QMM models under Bayesian and GTR under Maximum Likelihood frameworks. Even after removing the third position as well as codons for arginine (AGR and CGN), leucine (CTN and ATH), and serine (TCN and AGY), which can cause phylogenetic artifacts [44], we found the paraphyly of Anthozoa, with Medusozoa the sister taxon to Octocorallia (Additional file 2: Figure S2 and Additional file 3: Figure S3). Consequently, according to our analyses, mitochondrial protein genes support the clade [Octocorallia + Medusozoa] (Figure 2). In contrast, the amino acid composition of Hydrozoa and Discomedusae is similar (Additional file 4: Figure S4) and the Dayhoff recoding recovered Discomedusae as sister-group of Cubozoa (a reduced version of Acraspeda), although with low support, suggesting that the monophyly of the clade [Hydrozoa + Discomedusae] might be due to an amino acid composition artifact. In fact, preliminary analyses including the very fast evolving and compositionally biased Bilateria using the site-heterogeneous CATGTR model supported the unlikely grouping of Bilateria and Cubozoa (data not shown). This suggests that the use of a complex model of sequence evolution and of a rich taxon sampling is not sufficient to overcome all the systematic errors in the mitochondrial protein dataset. With the current increase in genome sequencing efforts, it will soon be possible to evaluate our phylogenetic tree (Figure 2) with large nuclear DNA datasets.

Within Medusozoa, Staurozoa is considered the first diverging clade, and the sister taxon to [Acraspeda (Cubozoa + Scyphozoa) + Hydrozoa] (reviewed in [2]). While the position of *Linuche unguiculata* is still ambiguous in our trees, we found no support for either the inclusion of coronates in Scyphozoa (PPs = 0; BV = 0), or for Acraspeda [Cubozoa + Scyphozoa] (PPs = 0; BV = 0). Instead, we found high support for the clades [Hydrozoa + Discomedusae] (PPs = 1; BV = 89) and [Cubozoa + Staurozoa] (CATGTR: PP = 0.83; GTR: PP = 1; BV = 58). This suggests that Scyphozoa is polyphyletic, although both Scyphozoa and Hydrozoa display similar amino acid composition. Additional sequences from coronates are needed to test the phylogenetic relationships presented here.

Recent molecular studies have refined our understanding of hydrozoan relationships, particularly the sister group relationship between Hydroidolina and Trachylina [7,14,18,19,30,51]. However, relationships within Hydroidolina have been very difficult to resolve based on either nuclear or mitochondrial rDNA genes. Here we have been able to sample five important hydroidolinan clades: Aplanulata, Capitata, Filifera III and IV, Leptothecata, and Limnomedusae. Mitochondrial protein genes provide good resolution for order-level relationships within Hydrozoa, although our sampling was limited in scope (only 10 species representing 6 out of 11 orders; Figure 2). We recovered monophyletic Hydroidolina as the sister group to our single representative from Trachylina. We also found monophyletic Aplanulata and Capitata, and paraphyletic Anthoathecata as suggested earlier [14,18]. On the other hand, we found a consistent grouping of capitate and aplanulate hydrozoans in all our trees (PPs = 1; BV = 88), which is in contradiction with earlier rDNA-based studies [7,18,19,29,30,49,51]. The high support values here suggest that higher resolution of the clade Hydroidolina may be achieved with an increase in the number of complete mtDNAs for representative taxa within this difficult clade.

Based on mitochondrial genome data, the monophyly of stony corals (Scleractinia) has recently been put into question [22], but more thorough studies employing alternative datasets have rejected this hypothesis [21,52]. Our analyses support the monophyly of Scleractinia under the preferred CATGTR model (PP = 0.51; AU = 0.25; KH = 0.17; SH = 0.53). Our phylogenetic analyses did not resolve relationships within octocorals. This was predictable given the low-level of variation of mitochondrial genes in octocorals (see [53]), a pattern attributed to the activity of the *mtMutS* gene they encode. It is noteworthy mentioning that our alignments did not encompass sequences from the *mtMutS* gene, absent in all other cnidarian and animal taxa, and which displays a comparatively higher rate of sequence evolution than other genes [54]. While future molecular studies of the evolutionary history of octocorals may focus their investigation to only a portion of the mtDNA [55], complete mitogenomes provide additional genomic features such as gene order and the composition of intergenic regions (IGRs) that could be valuable to systematic studies [10]. Furthermore, the combination of mitogenomic sequences with nuclear data will likely provide even better phylogenetic resolution for this group.

### Morphological evolution in cnidarians

**Figure 3:**
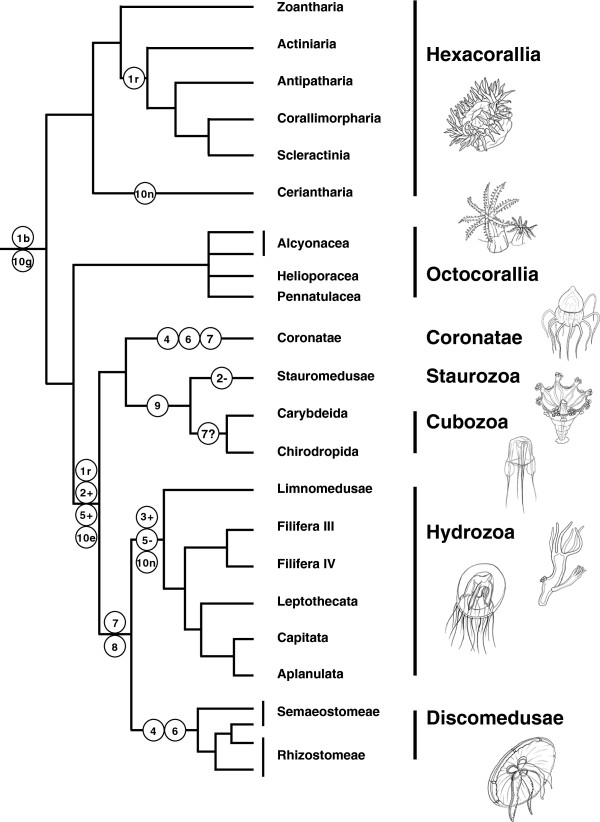
**Morphological character reconstruction in Cnidaria.** Reconstruction of key morphological characters in Cnidaria based on the phylogenetic hypothesis based on mitogenomic data. (1) symmetry: b = bilaterial, r = radial or biradial; (2) gain (+) and loss (−) of free-swimming medusoid stage (many hydrozoans have secondarily lost the medusoid phase); (3) velum (lost in some leptothecate hydrozoans); (4) strobilation; (5) gastric filaments; (6) ephyrae; (7) radial canal (scored uncertain in Cubozoa); (8) circular canal; (9) square symmetry of horizontal cross section; (10) gastrodermal muscles: e = in bunches of ectodermal origin, g = in bunches of gastrodermal origin, n = not organized in bunches. Morphological characters are taken from Marques and Collins (2004) and Cartwright and Nawrocki (2010) studies. Drawings were provided by Matthew Palen.

Unlike the dichotomous Anthozoa-Medusozoa, our strongly supported finding that Anthozoa is paraphyletic further supports the idea that bilateral symmetry, a step of foremost importance in metazoan evolution as it is exhibited as part of most animal body plans (Bauplan), was anciently acquired prior to the divergence between Cnidaria and Bilateria. In fact in Cnidaria, increasing evidence supports the presence of bilateral symmetry in corals and sea anemones, where it is represented by the siphonoglyph [56,57], while most medusozoans do not exhibit any bilateral symmetry [28,58], with siphonophores being an exception [59]. The tree topologies provided here strengthen the view that the ancestral cnidarian displayed a bilateral symmetry from which the radial tetrameral symmetry (body divided into four identical parts) of most medusae and many polyps of medusozoans derived (Figure 3). Previous studies have already suggested occurrences of deviation from a bilateral Bauplan in several animal groups, such as siphonophores [59], myxozoans [60] and some bilaterians [61,62]. Bilaterality has evolved very early in animal evolution near the root of the metazoan tree [63], and our data support the view that such a step was taken before the divergence of Cnidaria. Future studies that resolve the position of cnidarians within the metazoan tree of life will shed light on the origin of bilateral symmetry in animals.

Based upon the basal position of Staurozoa in previous phylogenies, Collins and collaborators [14] proposed that the medusa of Hydrozoa, Cubozoa and Scyphozoa derived from a stauromedusa-like ancestor. However, if the mitogenomic hypothesis of Cnidaria is true (Figure 3), then the free-living medusa form likely evolved in the branch leading to monophyletic Medusozoa, with subsequent independent losses of this life stage occurring in the lineages leading to Staurozoa and several hydrozoan clades (Figure 3). This scenario is consistent with earlier studies that originated the name "stalked jellyfish" for staurozoans, and concluded that these species represented "degenerated" jellyfish descended from ancestors with a pelagic medusa [14,16,64,65]. Simplification or losses of the medusa form has also been documented in several Hydrozoa clades [18]. The acquisition of a pelagic form is significant given that a free-swimming medusa allows a higher degree of offspring dispersion than by gametes and larvae alone [66].

Earlier studies have suggested several synapomorphies for the extended Acraspeda clade (Cubozoa + Scyphozoa + Staurozoa), namely radial tetrameral symmetry, medusa formation located at the apical end of the polyp, polyps with canal system and gastrodermal musculature organized in bunches of ectodermal origin (4 inter-radial within the mesoglea in Staurozoa and Scyphozoa, but not limited to 4 in Cubozoa), the presence of rhopalia or rhopalioid-like structures, medusae with gastric filaments and septa in the gastrovascular cavity [28,58]. Paraphyletic Acraspeda as suggested by our analyses implies that either these characters were acquired several times independently in Cubozoa, Coronatae and Discomedusae, or that they have been inherited from the ancestral medusozoan and lost in Hydrozoa. Given the complexity of these characters, it is unlikely that they were re-derived independently in various lineages. Rather, the most parsimonious scenario is their presence in the last common ancestor of medusozoans, with subsequent loss(es) in hydrozoans. Similarly, Scyphozoa are defined by the presence of ephyrae and simple rhopalia, and production of medusae through strobilation of the polyp [1,14,28,58]. If Scyphozoa are paraphyletic as suggested by our trees, these characters can be either convergent or plesiomorphic. For instance, rhopalia-like structures are present in all medusozoan clades but Hydrozoa. According to the mitogenomic view of cnidarian phylogeny, the most parsimonious scenario suggests that the ancestral medusozoan possessed some sort of rhopalium, maybe similar to those present in discomedusans and coronates. According to this scenario, simplification of rhopalia must have accompanied the degeneration of the medusae in Staurozoa, while they were completely lost in Hydrozoa. By contrast, the rhopalia in Cubozoa have evolved into complex structures with multiple, complex eyes. Marques and Collins (2004) have suggested a clade formed by [Cubozoa + Staurozoa] based on cladistic analysis of a set of morphological and life-history characters [28]. They suggested the presence of y-shaped septa and a quadrate or square symmetry of horizontal cross-section as synapomorphies for this clade. Mitogenomic data also support such a grouping, providing additional evidence for the validity of these characters as synapomorphies for the clade [Cubozoa + Staurozoa].

## Conclusions

We used an extended dataset of mitochondrial protein genes to reevaluate the phylogeny of Cnidaria, paying attention to common biases in phylogenetic reconstructions resulting from insufficient taxon sampling and using more simplistic models of sequence evolution. Our phylogenetic analyses suggest the grouping of Octocorallia and Medusozoa to the exclusion of Hexacorallia, resulting in paraphyletic Anthozoa. We also recovered the [Discomedusae + Hydrozoa] and [Coronatae + [Cubozoa + Staurozoa]] relationships within Medusozoa. It should be noted, however, that although our data provide little or no support for the clades Anthozoa, Acraspeda and Scyphozoa, they are not rejected with statistically significant support in the maximum-likelihood framework. Using the new mito-phylogenomic view, we reconstructed the evolution of several morphological characters in medusozoans. In particular, our phylogenetic hypothesis provided additional evidence for the “polyp first” theory, where the ancestral cnidarian was a bilateral polyp-like organism, and that a radially symmetrical and vagile medusa evolved in the branch leading to Medusozoa. Our analyses support the view that the ancestor of cnidarians and bilaterians (UrEumetazoa) possessed bilateral symmetry [67]. According to our working hypothesis, synapomorphies traditionally associated with Acraspeda such as the presence of gastric filaments in the medusae and gastrodermal musculature organized in bunches of ectodermal origin were most likely acquired early in the evolution of Medusozoa, and later lost in the branch leading to Hydrozoa. Finally, our study highlights some of the limitations of mt-based phylogenies and call for the concomitant use of several markers for resolving such deep nodes in the metazoan tree of life.

## Methods

### Mitochondrial sequence acquisition and alignment

We determined the complete mitochondrial genome sequences from the black coral *Cirripathes lutkeni* (NC_018377), the sea pens *Renilla muelleri* (NC_018378) and *Stylatula elongata* (NC_018380), the alcyonarian *Sinularia peculiaris* (NC_018379), as well as partial sequences from the cerianthid *Ceriantheopsis americanus* (JX023261-JX023265) and the octocoral *Heliopora coerulea* (JX023267-JX023272) as described previously [27]. In order to understand the phylogenetic signal contained within mitochondrial genomes, we constructed a dataset containing these new sequences, complete or nearly complete mitochondrial sequences from 24 medusozoan species that our group recently generated [27], as well as 51 other sequences previously available in GenBank, using four placozoan and 22 poriferan species (Table 3) as outgroup taxa. Our dataset covered most of the cnidarian diversity with representatives from all medusozoan classes, including cubozoans and staurozoans, which were only recently sampled [27]. The resulting dataset contains 106 taxa, 75 of which are cnidarian species, with representatives from the three Scyphozoa, the two Cubozoa and six of the 11 Hydrozoa orders.

**Table 3:** Species list

**Phylum**	**Subphylum**	**Class**	**Subclass**	**Order**	**Species**	**Accession number**	**Reference**
Cnidaria	Anthozoa	Hexacorallia		Actiniaria	*Metridium senile*	[GenBank:NC_000933]	[68]
					*Nematostella*sp	[GenBank:NC_008164]	[22]
				Antipatharia	*Chrysopathes formosa*	[GenBank:NC_008411]	[39]
					***Cirripathes lutkeni***	**[GenBank:NC_018377]**	**this study**
					*Leiopathes glaberrima*	[GenBank:FJ597643] & [GenBank:FJ597644]	[69]
				Corallimorpharia	*Discosoma*sp	[GenBank:NC_008071]	[22]
					*Rhodactis*sp	[GenBank:NC_008158]	[22]
					*Ricordea florida*	[GenBank:NC_008159]	[22]
				Scleractinia	*Acropora tenuis*	[GenBank:NC_003522]	[70]
					*Agaricia humilis*	[GenBank:NC_008160]	[22]
					*Anacropora matthai*	[GenBank:NC_006898]	Unpublished
					*Astrangia*sp	[GenBank:NC_008161]	[22]
					*Colpophyllia natans*	[GenBank:NC_008162]	[22]
					*Lophelia pertusa*	[GenBank:NC_015143]	[71]
					*Madracis mirabilis*	[GenBank:NC_011160]	[72]
					*Montastraea annularis*	[GenBank:NC_007224]	[73]
					*Montastraea faveolata*	[GenBank:NC_007226]	[73]
					*Montastraea franksi*	[GenBank:NC_007225]	[73]
					*Montipora cactus*	[GenBank:NC_006902]	Unpublished
					*Mussa angulosa*	[GenBank:NC_008163]	[22]
					*Pavona clavus*	[GenBank:NC_008165]	[22]
					*Pocillopora damicornis*	[GenBank:NC_009797]	[74]
					*Pocillopora eydouxi*	[GenBank:NC_009798]	[74]
					*Porites porites*	[GenBank:NC_008166]	[22]
					*Seriatopora caliendrum*	[GenBank:NC_010245]	[72]
					*Seriatopora hystrix*	[GenBank:NC_010244]	[72]
					*Siderastrea radians*	[GenBank:NC_008167]	[22]
					*Stylophora pistillata*	[GenBank:NC_011162]	[72]
				Zoantharia	*Palythoa*sp	[GenBank: DQ640650]	[22]
					*Savalia savaglia*	[GenBank:NC_008827]	[75]
				Ceriantharia	***Ceriantheopsis americanus***	**[GenBank:JX023261-JX023265]**	**this study**
		Octocorallia		Alcyonacea	*Acanella eburnea*	[GenBank:NC_011016]	[76]
					*Briareum asbestinum*	[GenBank:NC_008073]	[22]
					*Corallium konojoi*	[GenBank:NC_015406]	[77]
					*Dendronephthya castanea*	[GenBank: GU047877]	[10]
					*Dendronephthya gigantean*	[GenBank:NC_013573]	Unpublished
					*Dendronephthya mollis*	[GenBank: HQ694725]	[10]
					*Dendronephthya putteri*	[GenBank: HQ694726]	[10]
					*Dendronephthya suensoni*	[GenBank: GU047878]	[10]
					*Echinogorgia complexa*	[GenBank: HQ694727]	[10]
					*Euplexaura crassa*	[GenBank: HQ694728]	[10]
					*Keratoisidinae* sp	[GenBank:NC_010764]	[78]
					*Paracorallium japonicum*	[GenBank:NC_015405]	[77]
					*Pseudopterogorgia bipinnata*	[GenBank:NC_008157]	[22]
					*Sarcophyton glaucum*	[GenBank: AF064823] & [GenBank: AF063191]	[79,80]
					*Scleronephthya gracillimum*	[GenBank: GU047879]	[10]
					***Sinularia peculiaris***	**[GenBank:NC_018379]**	**this study**
				Helioporacea	***Heliopora coerulea***	**[GenBank:JX023267-JX023272]**	**this study**
				Pennatulacea	***Renilla mulleri***	**[GenBank:NC_018378]**	**this study**
					***Stylatula elongata***	**[GenBank:NC_018380]**	**this study**
	Medusozoa	Cubozoa		Carybdeida	*Alatina moseri*	[GenBank:JN700951-JN700958]	[27]
					*Carukia barnesi*	[GenBank:JN700959-JN700962]	[27]
					*Carybdea xaymacana*	[GenBank:JN700977-JN700983]	[27]
				Chirodropida	*Chironex fleckeri*	[GenBank:JN700963-JN700968]	[27]
					*Chiropsalmus quadrumanus*	[GenBank:JN700969-JN700974]	[27]
		Hydrozoa	Trachylina	Limnomedusae	*Cubaia Aphrodite*	[GenBank:JN700942]	[27]
			Hydroidolina	Aplanulata	*Ectopleura larynx*	[GenBank:JN700938]	[27]
					*Hydra magnipapillata*	[GenBank:NC_011220] & [GenBank:NC_01122]	[81]
					*Hydra oligactis*	[GenBank:NC_010214]	[8]
					*Hydra vulgaris*	[GenBank:BN001179] & [GenBank:BN001180]	[81]
				Capitata	*Millepora platyphylla*	[GenBank:JN700943]	[27]
					*Pennaria tiarella*	[GenBank:JN700950]	[27]
				Filifera III	*Clava multicornis*	[GenBank:JN700935]	[27]
				Filifera IV	*Nemopsis bachei*	[GenBank:JN700947]	[27]
				Leptothecata	*Laomedea flexuosa*	[GenBank:JN700945]	[27]
		Scyphozoa		Coronatae	*Linuche unguiculata*	[GenBank:JN700939]	[27]
			Discomedusae	Rhizostomeae	*Cassiopea Andromeda*	[GenBank:JN700934]	[27]
					*Cassiopea frondosa*	[GenBank:JN700936]	[27]
					*Catostylus mosaicus*	[GenBank:JN700940]	[27]
					*Rhizostoma pulmo*	[GenBank:JN700987] & [GenBank:JN700988]	[27]
				Semaeostomeae	*Aurelia aurita*A	[GenBank:NC_008446]	[11]
					*Aurelia aurita*B	[GenBank: HQ694729]	[10]
					*Chrysaora quinquecirrha*	[GenBank: HQ694730]	[10]
					*Chrysaora*sp	[GenBank:JN700941]	[27]
					*Cyanea capillata*	[GenBank:JN700937]	[27]
					*Pelagia noctiluca*	[GenBank:JN700949]	[27]
		Staurozoa		Stauromedusae	*Craterolophus convolvulus*	[GenBank:JN700975] & [GenBank:JN700976]	[27]
					*Haliclystus sanjuanensis*	[GenBank:JN700944]	[27]
					*Lucernaria janetae*	[GenBank:JN700946]	[27]
Placozoa					*BZ10101*	[GenBank:NC_008832]	[82]
					*BZ243*	[GenBank:NC_008834]	[82]
					*BZ49*	[GenBank:NC_008833]	[82]
					*Trichoplax adhaerens*	[GenBank:NC_008151]	[83]
Porifera		Homoscleromorpha			*Corticium candelabrum*	[GenBank:NC_014872]	[38]
					*Oscarella carmela*	[GenBank:NC_009090]	[84]
					*Plakina monolopha*	[GenBank:NC_014884]	[38]
					*Plakinastrella cf. onkodes*	[GenBank:NC_010217]	[84]
		Demospongiae	G1 = Keratosa		*Igernella notabilis*	[GenBank:NC_010216]	[84]
					*Ircinia strobilina*	[GenBank:NC_013662]	[85]
			G2 = Myxospongiae		*Aplysina fulva*	[GenBank:NC_010203]	[84]
					*Chondrilla aff. nucula*	[GenBank:NC_010208]	[84]
					*Halisarca dujardini*	[GenBank:NC_010212]	[84]
			G3 = marine Haplosclerida		*Amphimedon compressa*	[GenBank:NC_010201]	[84]
					*Callyspongia plicifera*	[GenBank:NC_010206]	[84]
					*Xestospongia muta*	[GenBank:NC_010211]	[84]
			G4 = Democlavia		*Agelas schmidti*	[GenBank:NC_010213]	[84]
					*Axinella corrugata*	[GenBank:NC_006894]	[84]
					*Cinachyrella kuekanthali*	[GenBank:NC_010198]	[84]
					*Ephydatia muelleri*	[GenBank:NC_010202]	[84]
					*Geodia neptuni*	[GenBank:NC_006990]	[37]
					*Lubomirskia baicalensis*	[GenBank:NC_013760]	[86]
					*Suberites domuncula*	[GenBank:NC_010496]	[87]
					*Tethya actinia*	[GenBank:NC_006991]	[37]
					*Topsentia ophiraphidites*	[GenBank:NC_010204]	[84]

List of species, with their accession number and reference, used for this studies. Bold species are from this study. Underline species are those included into the codon alignments.

### Sequence alignments

For this study, we focused our attention on the mitochondrial protein coding genes *atp6* and *8*, *cob*, *cox1-3*, *nad1-6* and *4L*. First, we created preliminary alignments of the amino acid sequences to correct potential frameshifts in our dataset. We then aligned the amino acid sequences of individual genes using the MUSCLE plug-in in Geneious Pro v5.5.6 [88] with default parameters, and we concatenated all the gene alignments into a single large dataset. We removed poorly aligned regions with Gblocks online (Castresana Lab, molevol.cmima.csic.es/castresana/) with the options allowing gap for all positions and 85% of the number of sequences for flanking positions. We manually checked the resulting alignment to correct for signs of frameshifts in sequences. The final alignment (AliMG) comprised 3485 amino acids (see Additional file 6).

In order to confirm our results from amino acid data, we also produced and analyzed several codon alignments. From the above 106 taxa list, we selected 75 taxa, including 10 octocorals and 20 hexacorals, to build several codon alignments. First, we create a codon alignment for each gene based on the concatenated amino acid alignment using the program PAL2NAL [89], before concatenating all genes into a single alignment (CodAliM75tx, 9921 parsimony-informative characters). We then created several additional codon alignments by removing the third codon position (CodAliM75tx-3, 5672 parsimony-informative characters); codons encoding for arginine (AGR and CGN) and leucine (CTN and ATH) (CodAliM75tx-argleu3, 5163 parsimony-informative characters); codons encoding for serine (TCN and AGY) (CodAliM75tx-ser3, 5318 parsimony-informative characters); and a combination of all three (CodAliM75tx-argleuser3, 4785 parsimony-informative characters). All the alignments are available upon request.

We used the program Net from the MUST package [90] to estimate the amino-acid composition per species in each of the alignments by assembling a 20 X 106 matrix containing the frequency of each amino acid. This matrix was then displayed as a two-dimensional plot in a principal component analysis, as implemented in the R package.

### Phylogenetic inferences

For the amino acid alignment AliMG, we conducted phylogenetic analyses under Maximum Likelihood (ML) and Bayesian (BI) frameworks using RAxML v7.2.6 and PhyloBayes v3.3 (PB), respectively [50,91-96]. PB analyses consisted of two chains over more than 11,000 cycles (maxdiff < 0.2) using CAT, GTR, and CAT + GTR models, and sampled every 10th tree after the first 100, 50 and 300 burn-in cycles, respectively for CAT, GTR and CAT + GTR. ML runs were performed for 1000 bootstrap iterations under the GTR model of sequence evolution with two parameters for the number of categories defined by a gamma (Γ) distribution and the CAT approximation. Under the ML framework, both analyses using the CAT approximation and Γ distribution of the rates across sites models yield nearly identical trees, suggesting that the GTR + CAT approximation does not interfere with the outcome of the phylogenetic runs for our dataset. In order to save computing time and power, we therefore opted for the CAT approximation with the GTR model for further tree search analyses under ML. We assessed the effect of missing data on cnidarian phylogenetic relationships in our trees by removing the partial sequences of *C. americanus* and *H. coerulea*. We also removed the coronate *Linuche unguiculata* given its problematic position and that it is the only representative of its clade, which could introduce a systematic bias. We then performed additional GTR analyses under the ML framework on the reduced, 103 taxa alignment.

We run jModelTest v2.0.2 [97] on all codon alignments to look for the models that best fit our data. We analyzed all the nucleotide alignments under both the BI framework using PhyloBayes v3.3 and MrBayes v3.2.1 (MB) [98] and ML framework using RAxML v7.2.6 as described above. For PB analyses we use the Q-Matrix Mixture model (QMM) instead of GTR and CAT + GTR + Γ. The MB analyses used the GTR + Γ + I model of sequence evolution and consisted of two chains of 5,000,000 generations, sampled every 1000th tree after the 25% burn-in.

### Evaluating phylogenetic inferences

We tested several relationships that had been earlier hypothesized with the amino acid dataset under ML by calculating the per-site log likelihood values with RAxML v7.2.6 under the GTR model. We then used three commonly used tests (the Approximately Unbiased (AU) test, the Kishino-Hasegawa (KH) test, and the Shimodaira-Hasegawa (SH) test) to assess the validity of several key phylogenetic relationships according to our data. To do so, we compared whether our data significantly rejected the best trees conforming to each of the *a priori* hypotheses (Table 2) using the CONSEL software [99].

For Bayesian inferences, a cross-validation was performed to find the model with the best fit to the data. Each alignment is randomly split in two unequal parts: a “learning dataset” with nine-tenth of the original positions and a “testing dataset” with one-tenth; ten replicates are performed. The parameters of the model are estimated on the learning datasets for each model (fixed topology inferred with the CATGTR model; 11,000 cycles; the first 1,000 cycles removed) and therefore used to calculate the cross-validation log-likelihood scores of the test datasets, averaged over the ten replicates.

Finally, we sampled ten important morphological characters from previously published morphological matrices [18,28,58,100] and mapped them on the CATGTR based tree. To do so we used PAUP 4.0b10 for Unix [101] using DELTRAN and ACCRAN character-state optimization models. The scoring of each character is detailed in additional materials (Additional file 7).

## Competing interests

The authors declare that they have no competing interests.

## Authors’ contributions

EK participated in the design of the study, carried out ML and BI phylogenetic analyses and statistical tests, reconstructed the evolution of morphological characters, and drafted the manuscript. BR carried out some of the PB phylogenetic analyses and extracted the support values, performed the cross validation. HP and DL helped in the design of the study. AGC provided guidance on some analyses and the interpretation of the morphological characters. All authors contributed to the final version of the manuscript.

## Supplementary Material

Additional file 1 **Figure S1.** Cnidarian phylogeny of mitochondrial protein genes using the reduced alignment AliMGred with 103 species. Phylogenetic analyses of cnidarian protein coding genes under the GTR model and CAT approximation with RAxML for the reduced AliMG alignment (AliMGred), where the coronate *Linuche unguiculata*, the tube anemone *Ceriantheopsis americanus* and the blue octocoral *Heliopora coerulea* were removed. Node supports correspond to the bootstraps values. Stars denote maximum support values. 1: Zoantharia; 2: Actiniaria; 3: Antipatharia; 4: Corallimorpharia; 5: Scleractinia; 6: Alcyonacea; 7: Pennatulacea; 8: Stauromedusae; 9: Carybdeida; 10: Chirodropida; 11: Limnomedusae; 12: Filifera III; 13: Filifera IV; 14: Leptothecata; 15: Capitata; 16: Aplanulata; 17: Semaeostomeae; 18: Rhizostomeae. Click here for file

Additional file 2 **Figure S2.** Cnidarian phylogeny of mitochondrial protein genes using the codon alignment CodAliM75tx-argleuser3. Phylogenetic analyses of cnidarian protein coding genes under the QMM + Γ model with PhyloBayes for the CodAliM75tx-argleuser3 alignments (4785 parsimony-informative characters). Support values correspond to the posterior probabilities for the QMM, the GTR(BI) and bootstraps for GTR(ML) analyses, respectively. Stars denote support values of PP > 0.98 and BV > 95. A dash denotes discrepancy between the results obtained by different methods. 1: Zoantharia; 2: Actiniaria; 3: Antipatharia; 4: Corallimorpharia; 5: Scleractinia; 6: Alcyonacea; 7: Pennatulacea; 8: Stauromedusae; 9: Carybdeida; 10: Chirodropida; 11: Limnomedusae; 12: Filifera III; 13: Filifera IV; 14: Leptothecata; 15: Capitata; 16: Aplanulata; 17: Semaeostomeae; 18: Rhizostomeae; 19: Ceriantharia; 20: Helioporacea. Click here for file

Additional file 3 **Figure S3.** Cnidarian phylogeny of mitochondrial protein genes using the codon alignment CodAliM75tx-ser3. Phylogenetic analyses of cnidarian protein coding genes under the QMM + Γ model with PhyloBayes for the CodAliM75tx-ser3 alignments (5318 parsimony-informative characters). Support values correspond to the posterior probabilities for QMM and GTR(BI) and bootstraps for GTR(ML) analyses, respectively. A star denotes support values of PP > 0.98 and BV > 95. A dash denotes discrepancy between the results obtained by different methods. 1: Zoantharia; 2: Actiniaria; 3: Antipatharia; 4: Corallimorpharia; 5: Scleractinia; 6: Alcyonacea; 7: Pennatulacea; 8: Stauromedusae; 9: Carybdeida; 10: Chirodropida; 11: Limnomedusae; 12: Filifera III; 13: Filifera IV; 14: Leptothecata; 15: Capitata; 16: Aplanulata; 17: Semaeostomeae; 18: Rhizostomeae; 19: Ceriantharia; 20: Helioporacea. Click here for file

Additional file 4 **Figure S4.** The use of several statistical tests verifying the validity of some groups in cnidarians for the reduced alignment AliMGred. Probability values for the AU, KH and SH tests and BI values for several clades traditionally recognized in Cnidaria for the reduced alignment AliMGred containing 103 species, where the coronate *Linuche unguiculata*, the tube anemone *Ceriantheopsis americanus* and the blue octocoral *Heliopora coerulea* were removed. Click here for file

Additional file 5 **Figure S5.** Composition of the amino acid alignment used in this study. Principal component analysis of the amino acid composition per species for the amino acid alignment AliMG used in this study. Species have been color-coded per group corresponding to each of the main cnidarian clades (Coronatae, Cubozoa, Discomedusa, Hexacorallia, Hydrozoa, Octocorallia, and Staurozoa), Porifera and Placozoa. The axes explain 33 and 22 per cent of the data. Click here for file

Additional file 6 **Figure S6.** Alignment AliMG. Alignments AliMG (3485 positions, 106 taxa) created using the combination MUSCLE + Gblocks. Click here for file

Additional file 7 **Figure S7.** Table of morphological characters mapped on the best tree. Mapping of morphological characters under DELTRAN and ACCTRAN models differed only for characters (5) and (7). (1) symmetry: medusozoan taxa have been scored radial, but Marques and Collins (2004) subdivided it into radial, biradial, or radial tetramerous; Octocorallia, Zoantharia and Ceriantharia are scored bilateral (Won et al. 2001), while the remnant of Hexacorallia was scored radial (Marques and Collins 2004); bilaterial symmetry is inferred the ancestral state for Cnidaira. (2) free-swimming medusoid stage: Hexa-, Octocorallia and Staurozoa all lack a free-living adult form (Marques and Collins 2004); the medusoid stage is also lacking in some Hydrozoa species but Collins (2002) and Cartwright and Nawrocki (2010) have inferred that the ancestral Hydrozoa had a medusoid stage. (3) velum: only present in Hydrozoa (Marques and Collins 2004). (4) strobilation: only described in Coronatae and Discomedusae (Marques and Collins 2004). (5) gastric filaments: we scored them as present in Cubozoa (Brigde et al. 1995), Staurozoa, Coronatae and Discomedusae (Marques and Collins 2004), and absent in Hydrozoa (Brigde et al. 1995), although suggested to be present in some Aplanulata (Bouillon et al. 2004); we decided to opt for the most parsimonious scenario. (6) ephyrae: only described in Coronatae and Discomedusae (Marques and Collins 2004). (7) radial canals: described in Coronatae, Discomedusae, and some Hydrozoa, absent in Staurozoa and unresolved in Cubozoa (Marques and Collins 2004); we choose two independent gains in Coronatae and Discomedusae as the most parsimonious scenario. (8) circular canals: present in Discomedusae and Hydrozoa, absent in the rest of Medusozoa. Marques and Collins (2004) have further distinguished the level of development of these structures that are partial in Discomedusae and full in Hydrozoa. (9) quadrate symmetry of horizontal cross section: present only in Cubozoa and Staurozoa (Marques and Collins 2004). (10) organization of the gastrodermal muscles of the polyp: organized in bunches of gastrodermic origin in all Hexacorallia but Ceriantharia, and inferred as the ancestral state for Cnidaria; organized in bunches of epidermic origin in all Medusozoa but Hydrozoa. In Hydrozoa and Ceriantharia, gastrodermal muscle are not organized in bunches (Marques and Collins 2004). Click here for file
