# Supplementary figures and images for: Cnidarian phylogenetic relationships as revealed by mitogenomics
Source: BMC Evol Biol. 2013 Jan 9;13:5. doi: 10.1186/1471-2148-13-5 (PMC3598815; doi:10.1186/1471-2148-13-5)

# Placozoa

# Porifera

## Hexacorallia

## Octocorallia

## 8 Staurozoa

## Cubozoa

## Hydrozoa

## Discomedusae

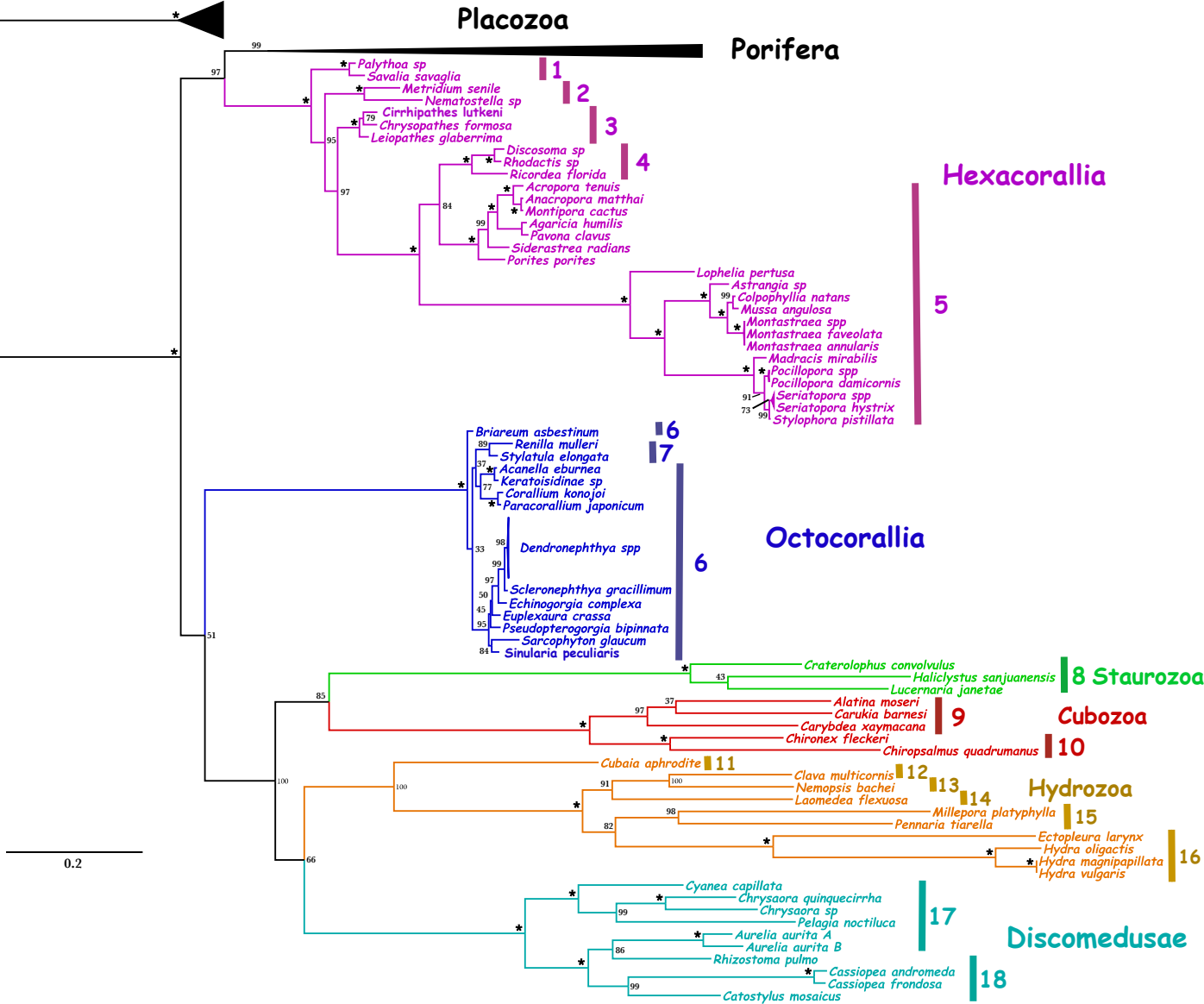

Supplement: Additional file 1 — Figure S1. Cnidarian phylogeny of mitochondrial protein genes using the reduced alignment AliMGred with 103 species. Phylogenetic analyses of cnidarian protein coding genes under the GTR model and CAT approximation with RAxML for the reduced AliMG alignment (AliMGred), where the coronate Linuche unguiculata, the tube anemone Ceriantheopsis americanus and the blue octocoral Heliopora coerulea were removed. Node supports correspond to the bootstraps values. Stars denote maximum support values. 1: Zoantharia; 2: Actiniaria; 3: Antipatharia; 4: Corallimorpharia; 5: Scleractinia; 6: Alcyonacea; 7: Pennatulacea; 8: Stauromedusae; 9: Carybdeida; 10: Chirodropida; 11: Limnomedusae; 12: Filifera III; 13: Filifera IV; 14: Leptothecata; 15: Capitata; 16: Aplanulata; 17: Semaeostomeae; 18: Rhizostomeae. [file 1471-2148-13-5-S1.pdf]

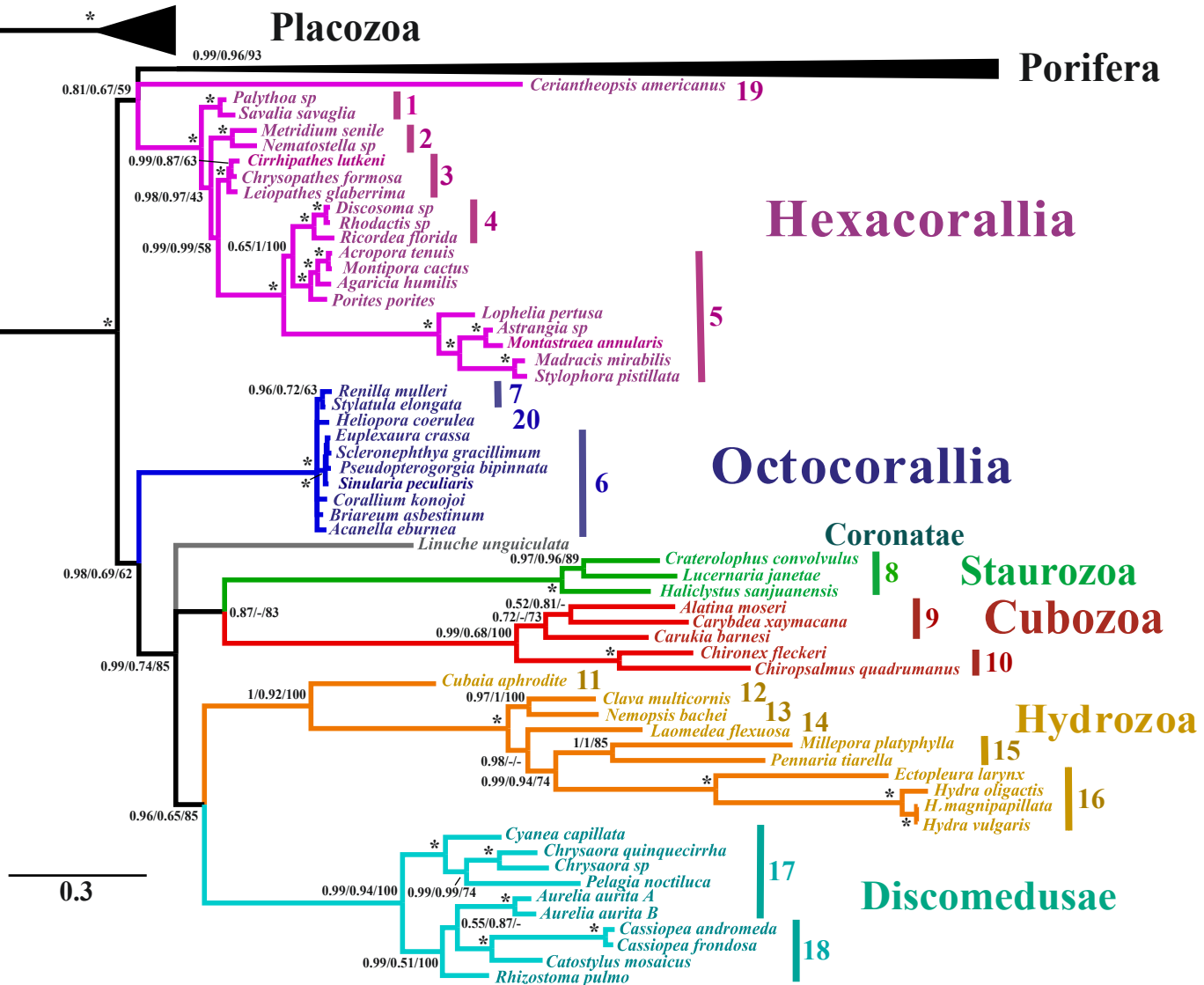

Supplement: Additional file 2 — Figure S2. Cnidarian phylogeny of mitochondrial protein genes using the codon alignment CodAliM75tx-argleuser3. Phylogenetic analyses of cnidarian protein coding genes under the QMM + Γ model with PhyloBayes for the CodAliM75tx-argleuser3 alignments (4785 parsimony-informative characters). Support values correspond to the posterior probabilities for the QMM, the GTR(BI) and bootstraps for GTR(ML) analyses, respectively. Stars denote support values of PP > 0.98 and BV > 95. A dash denotes discrepancy between the results obtained by different methods. 1: Zoantharia; 2: Actiniaria; 3: Antipatharia; 4: Corallimorpharia; 5: Scleractinia; 6: Alcyonacea; 7: Pennatulacea; 8: Stauromedusae; 9: Carybdeida; 10: Chirodropida; 11: Limnomedusae; 12: Filifera III; 13: Filifera IV; 14: Leptothecata; 15: Capitata; 16: Aplanulata; 17: Semaeostomeae; 18: Rhizostomeae; 19: Ceriantharia; 20: Helioporacea. [file 1471-2148-13-5-S2.pdf]

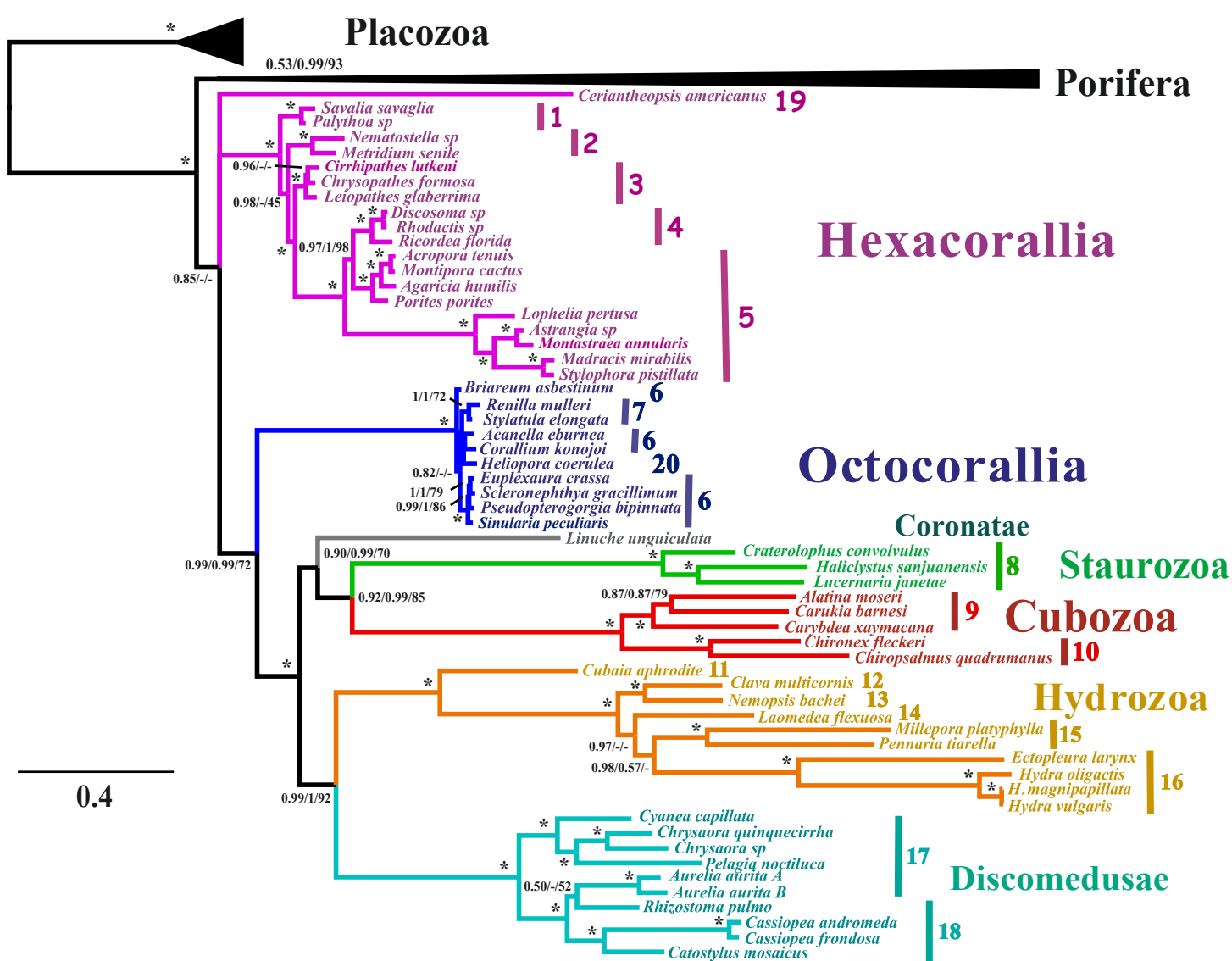

Supplement: Additional file 3 — Figure S3. Cnidarian phylogeny of mitochondrial protein genes using the codon alignment CodAliM75tx-ser3. Phylogenetic analyses of cnidarian protein coding genes under the QMM + Γ model with PhyloBayes for the CodAliM75tx-ser3 alignments (5318 parsimony-informative characters). Support values correspond to the posterior probabilities for QMM and GTR(BI) and bootstraps for GTR(ML) analyses, respectively. A star denotes support values of PP > 0.98 and BV > 95. A dash denotes discrepancy between the results obtained by different methods. 1: Zoantharia; 2: Actiniaria; 3: Antipatharia; 4: Corallimorpharia; 5: Scleractinia; 6: Alcyonacea; 7: Pennatulacea; 8: Stauromedusae; 9: Carybdeida; 10: Chirodropida; 11: Limnomedusae; 12: Filifera III; 13: Filifera IV; 14: Leptothecata; 15: Capitata; 16: Aplanulata; 17: Semaeostomeae; 18: Rhizostomeae; 19: Ceriantharia; 20: Helioporacea. [file 1471-2148-13-5-S3.pdf]

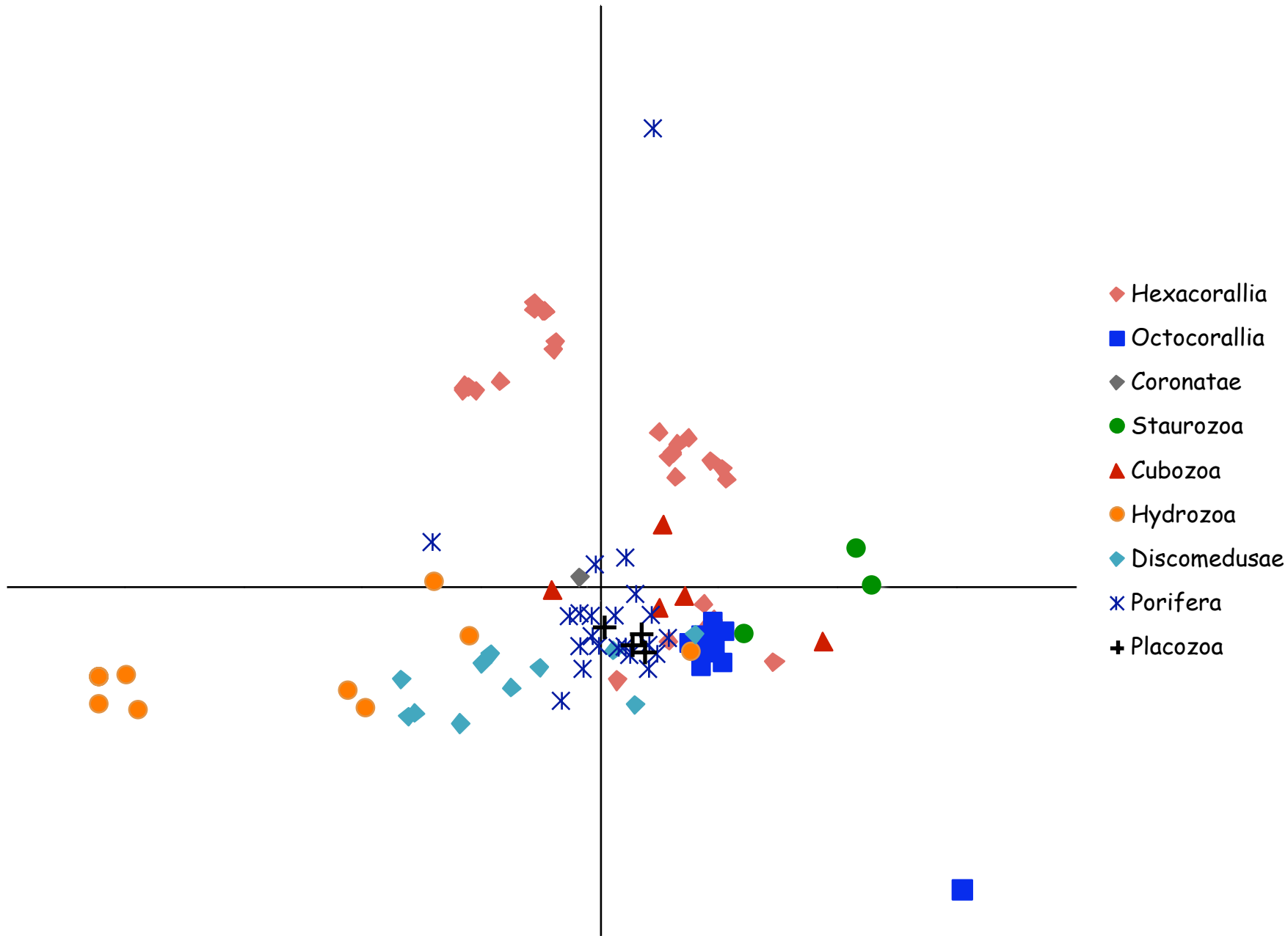

Supplement: Additional file 5 — Figure S5. Composition of the amino acid alignment used in this study. Principal component analysis of the amino acid composition per species for the amino acid alignment AliMG used in this study. Species have been color-coded per group corresponding to each of the main cnidarian clades (Coronatae, Cubozoa, Discomedusa, Hexacorallia, Hydrozoa, Octocorallia, and Staurozoa), Porifera and Placozoa. The axes explain 33 and 22 per cent of the data. [file 1471-2148-13-5-S5.pdf]
