# Supplementary material for: Cnidarian phylogenetic relationships as revealed by mitogenomics
Source: BMC Evol Biol. 2013 Jan 9;13:5. doi: 10.1186/1471-2148-13-5 (PMC3598815; doi:10.1186/1471-2148-13-5)
Supplement: Additional file 4 — Figure S4. The use of several statistical tests verifying the validity of some groups in cnidarians for the reduced alignment AliMGred. Probability values for the AU, KH and SH tests and BI values for several clades traditionally recognized in Cnidaria for the reduced alignment AliMGred containing 103 species, where the coronate Linuche unguiculata, the tube anemone Ceriantheopsis americanus and the blue octocoral Heliopora coerulea were removed. [file 1471-2148-13-5-S4.pdf]

	AliMGred		
	AU	KH	SH
Acraspeda	0.75	0.66	0.90
Scyphozoa + Cubozoa + Hydrozoa*	0.23	0.22	0.39
Scyphozoa + Cubozoa + Staurozoa*	0.08	0.18	0.26
Scyphozoa + Staurozoa	0.02	0.02	0.20
