# Supplementary material for: Cnidarian phylogenetic relationships as revealed by mitogenomics
Source: BMC Evol Biol. 2013 Jan 9;13:5. doi: 10.1186/1471-2148-13-5 (PMC3598815; doi:10.1186/1471-2148-13-5)
Supplement: Additional file 7 — Figure S7. Table of morphological characters mapped on the best tree. Mapping of morphological characters under DELTRAN and ACCTRAN models differed only for characters (5) and (7). (1) symmetry: medusozoan taxa have been scored radial, but Marques and Collins (2004) subdivided it into radial, biradial, or radial tetramerous; Octocorallia, Zoantharia and Ceriantharia are scored bilateral (Won et al. 2001), while the remnant of Hexacorallia was scored radial (Marques and Collins 2004); bilaterial symmetry is inferred the ancestral state for Cnidaira. (2) free-swimming medusoid stage: Hexa-, Octocorallia and Staurozoa all lack a free-living adult form (Marques and Collins 2004); the medusoid stage is also lacking in some Hydrozoa species but Collins (2002) and Cartwright and Nawrocki (2010) have inferred that the ancestral Hydrozoa had a medusoid stage. (3) velum: only present in Hydrozoa (Marques and Collins 2004). (4) strobilation: only described in Coronatae and Discomedusae (Marques and Collins 2004). (5) gastric filaments: we scored them as present in Cubozoa (Brigde et al. 1995), Staurozoa, Coronatae and Discomedusae (Marques and Collins 2004), and absent in Hydrozoa (Brigde et al. 1995), although suggested to be present in some Aplanulata (Bouillon et al. 2004); we decided to opt for the most parsimonious scenario. (6) ephyrae: only described in Coronatae and Discomedusae (Marques and Collins 2004). (7) radial canals: described in Coronatae, Discomedusae, and some Hydrozoa, absent in Staurozoa and unresolved in Cubozoa (Marques and Collins 2004); we choose two independent gains in Coronatae and Discomedusae as the most parsimonious scenario. (8) circular canals: present in Discomedusae and Hydrozoa, absent in the rest of Medusozoa. Marques and Collins (2004) have further distinguished the level of development of these structures that are partial in Discomedusae and full in Hydrozoa. (9) quadrate symmetry of horizontal cross section: present only in Cubozoa and S [file 1471-2148-13-5-S7.pdf]

	1	2	3	4	5	6	7	8	9	10
Actiniaria	1	0	0	0	0	0	0	0	0	1
Antipatharia	1	0	0	0	0	0	0	0	0	1
Corallimorpharia	1	0	0	0	0	0	0	0	0	1
Scleractinia	1	0	0	0	0	0	0	0	0	1
Zoantharia	0	0	0	0	0	0	0	0	0	1
Ceriantharia	0	0	0	0	0	0	0	0	0	2
Alcyonacea	0	0	0	0	0	0	0	0	0	1
Helioporacea	0	0	0	0	0	0	0	0	0	1
Pennatulacea	0	0	0	0	0	0	0	0	0	1
Carybdeida	1	1	0	0	1	0	?	0	1	0
Chirodropida	1	1	0	0	1	0	?	0	1	0
Limnomedusae	1	1	1	0	0	0	1	1	0	2
Aplanulata	1	1	1	0	0	0	1	1	0	2
Capitata	1	1	1	0	0	0	1	1	0	2
Filifera III	1	1	1	0	0	0	1	1	0	2
Filifera IV	1	1	1	0	0	0	1	1	0	2
Leptothecata	1	1	1	0	0	0	1	1	0	2
Coronatae	1	1	0	1	1	1	1	0	0	0
Rhizostomeae	1	1	0	1	1	1	1	1	0	0
Semaeostomeae	1	1	0	1	1	1	1	1	0	0
Stauromedusae	1	0	0	0	1	0	0	0	1	0
